# *ARNT* is a potential direct HIF-1 target gene in human Hep3B hepatocellular carcinoma cells

**DOI:** 10.1186/s12935-017-0446-2

**Published:** 2017-08-24

**Authors:** Markus Mandl, Reinhard Depping

**Affiliations:** 1 Institute of Physiology, Center for Structural and Cell Biology in Medicine, University of Luebeck, Ratzeburger Allee 160, 23562 Lübeck, Germany; 2 Division of Cell Metabolism and Differentiation Research, Institute for Biomedical Aging Research, University of Innsbruck, Rennweg 10, 6020 Innsbruck, Austria

**Keywords:** Aryl hydrocarbon receptor nuclear translocator, Oxygen sensing, Hep3B, HIF-1b, Hypoxia

## Abstract

**Background:**

The transcription factor aryl hydrocarbon receptor nuclear translocator (ARNT) participates in the hypoxia-inducible factor (HIF) pathway which senses a decline in cellular oxygen tension. In hypoxia, HIF-1α and ARNT form the transcriptional active complex HIF-1 followed by the expression of target genes. ARNT is considered as constitutively expressed and unaffected by hypoxia. However, certain tumour cell lines derived from different entities are capable to elevate ARNT expression under hypoxic conditions which implies a survival benefit. It was demonstrated that high ARNT protein levels mediate radioresistance in tumour cells. Furthermore, a HIF-1α-driven feed-forward loop leading to augmented HIF signalling was discovered in Hep3B cells. Herein HIF-1α elevates the mRNA and protein expression of its binding partner ARNT in hypoxia. However, the detailed mechanism remained unclear. The objective of this study was to test whether HIF-1α might directly regulate ARNT expression by recruitment to the *ARNT* promoter.

**Methods:**

Chromatin immunoprecipitation (ChIP), CRISPR/Cas9 genome editing, Western blotting, quantitative RT-PCR and reporter gene assays were applied. The unpaired *t* test was used for statistical analysis.

**Results:**

ChIP assays revealed the binding of both HIF-1α and ARNT to the *ARNT* promoter in hypoxia. The relevance of this particular region for hypoxic ARNT induction was confirmed by CRISPR/Cas9 genome editing. ARNT normoxic basal expression and hypoxic inducibility was reduced in genome-edited Hep3B cells. This phenotype was accompanied with impaired HIF signalling and was rescued by ARNT overexpression.

**Conclusions:**

The results indicate *ARNT* to be a putative HIF-1 target gene and a limiting factor in this model.

**Electronic supplementary material:**

The online version of this article (doi:10.1186/s12935-017-0446-2) contains supplementary material, which is available to authorized users.

## Introduction

Members of the basic helix-loop-helix Per-ARNT-Sim (bHLH-PAS) family of transcription factors play pivotal roles in several signal transduction pathways [1]. Moreover, one factor can act within different signalling circuits thus leading to crosstalk. Both terms apply for the transcription factor aryl hydrocarbon receptor nuclear translocator (ARNT) which is also designated as hypoxia-inducible factor (HIF)-1β [1, 2]. ARNT interconnects the HIF and the aryl hydrocarbon receptor (AhR) pathways which sense a decline in oxygen tension (hypoxia) or the presence of xenobiotics (i.e., dioxins) respectively [1, 2].

In general, bHLH-PAS proteins need to form heterodimers in order to become transcriptional active complexes. Activation of a signal-regulated subunit (i.e., class I bHLH-PAS protein) triggers its translocation into the cell nucleus and enables heterodimerisation with another required family member (i.e., class II bHLH-PAS protein; e.g., ARNT) [1]. Within the HIF pathway, HIF-1α is the predominant and best characterised subunit. Under normoxic conditions (i.e., sufficient oxygen supply), HIF-1α is hydroxylated at two conserved proline residues by prolyl hydroxylase domain (PHD) enzymes. Subsequently the tumour suppressor protein *von Hippel*–*Lindau* (pVHL), which is part of an ubiquitin ligase complex, recognises this post-translational modification and triggers proteasomal degradation. In hypoxia, PHD enzymes are inhibited leading to HIF-1α accumulation and nuclear translocation [2, 3]. Inside this cellular compartment, HIF-1α and ARNT heterodimerise and form the transcriptional active complex HIF-1. Expression of HIF target genes is initiated in conjunction with co-factors such as CBP/p300 [2]. HIF induced genes are characterised by the presence of a hypoxia-responsive element (HRE) within the promoter or enhancer region [4]. This element consists of the consensus sequence 5′-RCGTG-3′ which is the minimal sequence required for HIF-1 binding (generally designated as HIF-1 binding site, HBS) [4, 5]. Moreover, the majority of HREs also contain a HIF-1 ancillary sequence (HAS) which is located in close proximity up- or downstream of the HBS and represents an imperfect inverted repeat of the HBS sequence [4]. Therefore, it was proposed that the secondary structure of HREs is crucial for its activator function [4].

On the other hand, the AhR pathway becomes activated by a wide range of xenobiotics derived from natural and industrial sources. These chemical compounds act as AhR ligands and enable nuclear translocation of the receptor. Inside the nucleus, AhR binds to ARNT and triggers the expression of target genes responsible for detoxification. In addition, there is evidence that the AhR pathway plays a crucial role in development [1]. AhR regulated genes are characterised by the presence of a xenobiotic-responsive element (XRE) [1, 2]. Moreover, the XRE consensus sequence 5′-TNGCGTG-3′ shares some similarities with the HRE [1].

In contrast to class I Per-ARNT-Sim transcription factors, the regulation of ARNT is less investigated. According to the general point of view, mentioned in the literature, ARNT is considered to be constitutively expressed [2]. This means that ARNT expression is not influenced by environmental conditions such as hypoxia. However, there is increasing evidence from several studies that tumour cells derived from different entities are capable to upregulate ARNT under oxygen deprivation [2, 6–10] (reviewed in Ref. [2]). Recently, we were able to elucidate cellular advantages of an elevated ARNT expression. ARNT overexpression in tumour cells conferred radioresistance whereas knockdown of *ARNT* had the opposite effect [11]. In addition, we recently discovered that hypoxic ARNT induction is part of a feed-forward loop in human hepatocellular carcinoma Hep3B cells [12]. This network motif consists of two transcription factors wherein one of them regulates the other and both control a target gene together. Herein, HIF-1α mediates the upregulation of its binding partner ARNT in hypoxia which augments HIF signalling. This regulatory relationship was shown on both mRNA and protein levels [12]. Noteworthy, such a non-canonical regulation of ARNT by HIF-1α was also demonstrated in another cell line [9].

Given that Hep3B cells show a pronounced induction of ARNT in hypoxia and are a widely used model in HIF biology, the aim of this study was to investigate whether HIF-1α might induce ARNT expression directly by binding to the *ARNT* gene promoter.

## Materials and methods

### Cell culture and hypoxic conditions

Human hepatocellular carcinoma Hep3B cells (ATCC) were maintained in RPMI 1640 medium (Gibco) containing 10% FBS and Penicillin/Streptomycin. Cell cultures were kept under standard normoxic conditions (21% O_2_) at 37 °C in a humidified atmosphere with 5% CO_2_. Cells were harvested by trypsination and subcultured in a ratio of 1:5–1:10.

For hypoxic exposure cells were incubated at 37 °C in a humidified atmosphere with 3% O_2_, 5% CO_2_ and balanced N_2_ for 5 or 8 h depending on type/purpose of the experiment.

### Chromatin immunoprecipitation (ChIP)

ChIP assays were conducted using the SimpleChIP^®^ Enzymatic Chromatin IP Kit (#9002, Cell Signaling Technology) according to the manufacturer’s protocol. Briefly, 5 × 10^6^ cells were seeded in 15 cm Petri-dishes and allowed to adhere overnight. Subsequently the supernatant was replaced by 25 ml fresh medium and cells were exposed to normoxia or hypoxia (3% O_2_) for 5 h. Crosslinking of chromatin was achieved by addition of 37% formaldehyde (1% final concentration) followed by an incubation on ice and room temperature for 5 min respectively. Immunoprecipitation was carried out using specific antibodies as listed in Table 1. A total amount of 2 µg antibody per sample was deployed. Co-precipitated DNA was purified using appropriate spin-columns (provided in the kit or by using the GeneJet™ Gel Extraction Kit (#K0692, Fermentas) respectively). Genomic DNA sequences were analysed in triplicates by qPCR (ABI Prism 7000, Applied Biosystems) using appropriate primers (Table 1) and SYBR green chemistry (FastStart Universal SYBR Green Master (Rox), Roche). An annealing temperature of 65 °C was used. C_t_ values obtained were normalised to the IgG isotype control and expressed as fold enrichment.

**Table 1:** Materials

Antibodies	Description	Application
Anti-ARNT	Mouse monoclonal, clone 2B10, #NB300-525, Novus Biologicals	ChIP, WB
Anti-HIF-1α	Mouse monoclonal, clone H1α67, #NB100-123, Novus Biologicals	ChIP
Anti-HIF-1α	Mouse monoclonal, clone 54, #610959, BD Transduction Laboratories™	WB
Anti-histone H3	Rabbit monoclonal, clone D2B12, #4620, Cell Signaling Technology	ChIP
Anti-Lamin A/C	Goat polyclonal, #sc-6215, Santa Cruz	WB
IgG	Isotype control, rabbit, #2729, Cell Signaling Technology	ChIP

Oligos	Sequence	
Target 1 top	GATTACAGGCATGCGCCACCACGCCGTTTT	
Target 1 bottom	GGCGTGGTGGCGCATGCCTGTAATCCGGTG	
Target 2 top	TTCGAACCCCTGGCCACAGGTGATCGTTTT	
Target 2 bottom	GATCACCTGTGGCCAGGGGTTCGAACGGTG	

Primer	Sequence	
Region 1 for	CAACGTCGTGAAACTCCATC	
Region 1 rev	TGCCTCAGTCTCCTGAGTAG	
Region 2 for	ACGGAGTTTCGCTCTTGTTG	
Region 2 rev	CCTGTAATCCCAGCTTCTTG	
Region 3 for	TGCCTCAGCCTCCCAAGAAG	
Region 3 rev	CGCGTCTGTAATCCCAGCAC	
RPL30	#7014, Cell Signaling Technology	
U6 sequencing	GGACTATCATATGCTTACCG	
VEGF HRE for	CAGTTCCCTGGCAACATCTG	
VEGF HRE rev	CAGTGACTGGGAGGGAAGAG	

### CRISPR/Cas9 genome editing

All-in-one CRISPR/Cas9 plasmids were generated using the GeneArt^®^ CRISPR Nuclease Vector Kit (#A21174, Life Technologies) as described in the supplier’s protocol. Sequences of oligonucleotides used as insert are given in Table 1. Finally, CRISPR/Cas9 constructs were confirmed by sequencing.

### Transient transfection

Plasmid transfection was performed using GeneJuice^®^ (Merck Millipore) in accordance with the manufacturer’s protocol. For transfection of CRISPR/Cas9 plasmids, cells were plated at a density of 2.5 × 10^5^ cells/well in 6-well plates using antibiotic-free medium and allowed to adhere overnight. Subsequently, cells were transfected with 3 µg plasmid DNA per well. A ratio of 1 µg DNA:5 µl GeneJuice^®^ was applied for all transfections. Cell number and amounts were adjusted accordingly for different culture vessels. After overnight incubation, the transfection mixture was replaced by fresh antibiotic-free medium and cells were exposed to normoxia or hypoxia depending on type/purpose of the experiment.

### Cleavage detection assays

The presence of genomic insertions or deletions (indels) within the selected locus was determined by the GeneArt^®^ Genomic Cleavage Detection Kit (#A24372, Life Technologies) as described in the manufacturer’s guidelines. Subsequently, PCR products were dissolved on a 2.5% agarose gel containing ethidium bromide and documented.

### Western blot analysis

Western blotting was carried out as described previously [11, 12]. Whole cell extracts (50 µg/lane) were dissolved on 7.5% acrylamide gels and transferred onto Polyvinyl difluoride (PVDF) membranes (Immobilon-P, 0.45 µm, Merck Millipore). Primary antibodies (Table 1) were diluted 1:1000 (except anti-ARNT 1:2000) and applied overnight. Determination of Lamin A/C was done for normalisation and the appropriate antibody was applied for 1 h at room temperature. Afterwards PVDF membranes were incubated with HRP-conjugated secondary antibodies (1:5000, DAKO) for 1 h at room temperature. Chemoluminescence development was achieved using the ECL reagent (Clarity™ Western ECL, Bio-Rad). Subsequently membranes were exposed to X-ray films (Amersham Hyperfilm MP, GE Healthcare) and signals were quantified using the AIDA Image Analyzer (Version 4.27, Raytest).

### Gene expression analysis

Expression of genes was analysed as described previously [11]. Briefly, mRNA expression was determined using TaqMan^®^ Gene Expression Assays (ARNT #Hs01121918_m1, HIF1A #Hs00936368_m1, VEGFA #Hs00900055_m1, Applied Biostems) and normalised to Beta-2-microglobulin (B2 M) mRNA (#Hs00187842_m1, Applied Biosystems). Real-time PCR was carried out with an ABI PRISM^®^ 7000 system (Applied Biosystems) using the protocol for comparative relative quantitation (∆∆C_t_ method).

### Reporter gene assays

Luciferase reporter gene assays were performed as described previously [12]. Briefly, 4 × 10^4^ cells/well were seeded in 24-well plates in antibiotic-free medium and incubated overnight. Subsequently, cells were co-transfected with the hypoxia-inducible *Firefly* luciferase construct (100 ng/well) and a constitutive *Renilla* luciferase expression vector (100 ng/well) for normalisation. In addition, cells were transfected with CRISPR/Cas9 plasmids as described above and treated depending on type/purpose of experiment. Luciferase expression was measured using the Dual-Luciferase Reporter Assay System (#E1960, Promega) according to the supplier’s instructions.

### Statistical analysis

Experimental results were statistically analysed using GraphPad Prism^®^ 4 software (GraphPad). All values are presented as mean ± SEM. Each experiment was performed independently at least three times. Comparison between two experimental groups was done using the unpaired *t* test. *P* values ≤0.05 were regarded as statistically significant.

## Results

### HIF-1α and ARNT are recruited to the *ARNT* promoter in hypoxia

**Fig. 1:**
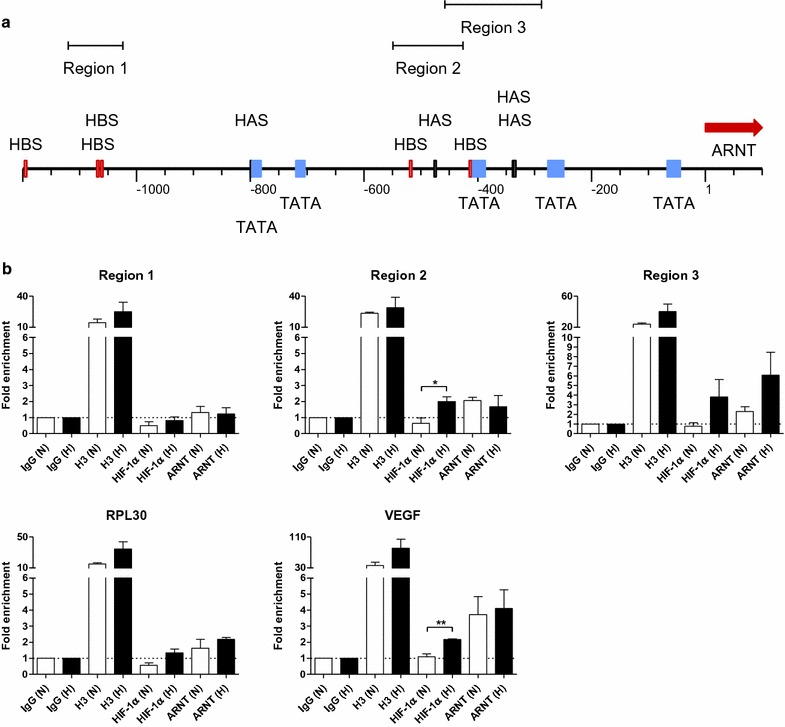
Recruitment of HIF-1α and ARNT to the *ARNT* gene promoter. **a** Functional elements within the *ARNT* promoter sequence upstream to −1200 bp from the transcription start site (bp 1). *HBS* HIF binding site, *HAS* HIF ancillary sequence, *TATA* TATA box. Investigated partial sequences are indicated (Region 1, Region 2, Region 3). **b** Chromatin immunoprecipitation (ChIP) assays. Values are presented as mean ± SEM of n = 3 independent experiments. *IgG* normal rabbit IgG, *H3* histone H3

The appropriate *ARNT* promoter sequence ranging from −1200 bp upstream to +100 bp downstream of the transcription start site (TSS) was retrieved from the Eukaryotic Promoter Database [13] (http://epd.vital-it.ch/). Subsequently, the *ARNT* promoter was screened for the presence of HIF binding sites (HBS) and HIF ancillary sequences (HAS) as defined in Ref. [4]. As shown in Fig. 1a, several HBS and HAS elements were found. In order to detect a putative HIF-1α binding event suitable primer pairs for chromatin immunoprecipitation (ChIP) were designed which flank the regulatory elements in question (Fig. 1a: Region 1, Region 2, Region 3). Specific antibodies against HIF-1α and its binding partner ARNT were used to pull down the appropriate transcription factors and cross-linked chromatin. In addition, histone H3 was precipitated for positive control. An IgG isotype control was used for normalisation. As shown in Fig. 1b, HIF-1α was not detected within Region 1. The ARNT signal obtained from this sequence was slightly above background. Different in Region 2, a significant enrichment of HIF-1α was found under hypoxic conditions. In line with this observation ARNT was also detected at this locus in normoxia and hypoxia. Remarkably, the highest enrichment of HIF-1α and ARNT was found in Region 3 under oxygen deprivation.

The gene locus of ribosomal protein L30 (RPL30) was used as a technical positive control to ensure the accuracy of ChIP assays. Herein no binding of HIF-1α was detected whereas ARNT was slightly enriched at this site. Moreover, the occupancy of the VEGF hypoxia-responsive element by HIF-1α and ARNT was tested too. As expected, HIF-1α was significantly enriched at this locus in hypoxia. A high enrichment of ARNT was also detected at this site even under normoxic conditions.

Taken together, these results demonstrate the recruitment of HIF-1α and ARNT to the *ARNT* promoter in hypoxia. The detection of ARNT at certain loci in normoxia indicates that ARNT binding is not essentially dependent on HIF-1α.

### Targeting of transcription factor binding sites by CRISPR/Cas9 genome editing

**Fig. 2:**
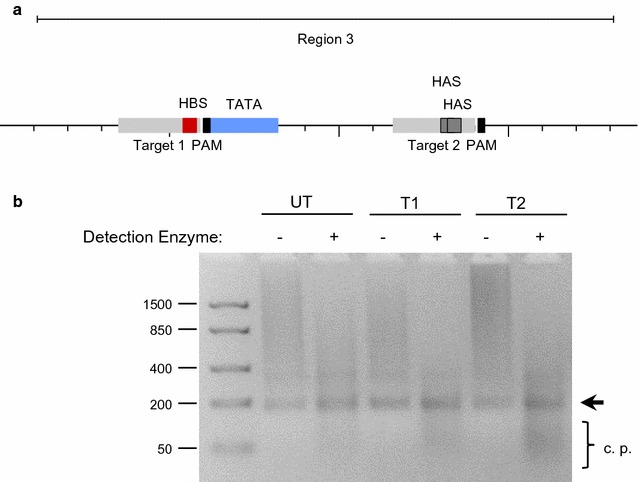
CRISPR/Cas9 genome editing of the *ARNT* gene promoter. **a** Location of CRISPR/Cas9 target sequences (*light grey*) within Region 3. *HBS* HIF binding site, *HAS* HIF ancillary sequence, *PAM* protospacer-adjacent motif. **b** Cleavage detection assay of untransfected (UT), CRISPR/Cas9-Target 1 (T1) or CRISPR/Cas9-Target 2 (T2) transfected Hep3B cells. Fragment sizes are given in bp. The *arrow* indicates the re-hybridised PCR product. Representative result of n = 3 independent experiments. *c. p.* cleavage products

In order to elucidate the importance of HIF-1α and ARNT recruitment to the *ARNT* gene promoter for hypoxia-inducible ARNT expression CRISPR/Cas9 genome editing was employed. Due to the high enrichment of both transcription factors in Region 3 appropriate CRISPR/Cas9 targets within this sequence were investigated using a web based tool [14] (http://www.rgenome.net/cas-designer/). Therefore, two different CRISPR/Cas9 target sites were selected. The sequence designated as Target 1 covers the HBS upstream of the TATA box but might be error prone due to numerous similarities across the genome. Target 2 encompass both HAS elements and was predicted to act with high fidelity due to only one putative off target effect (Fig. 2a; Additional file 1: Figure S1).

The ability to create insertions or deletions (indels) by the appropriate CRISPR/Cas9 constructs within Region 3 was tested by a cleavage detection assay. As shown in Fig. 2b, the appearance of cleavage products below the size of the re-hybridised PCR fragment indicates the presence of mutations. Such genomic alterations were mainly detected in CRISPR/Cas9-Target 2 transfected Hep3B cells.

### CRISPR/Cas9 genome editing of a highly HIF-1α and ARNT enriched locus within the *ARNT* promoter sequence impairs hypoxia-dependent ARNT upregulation

**Fig. 3:**
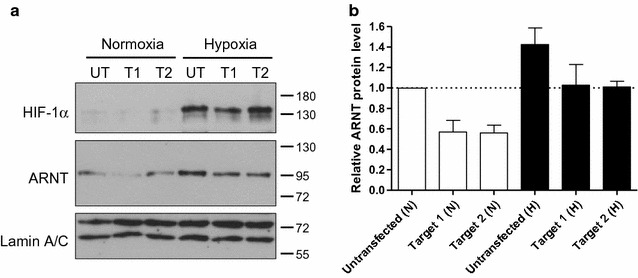
Western blot analysis of CRISPR/Cas9 genome edited Hep3B cells. **a** Representative Western blot of n = 4 independent experiments. Untransfected (UT), CRISPR/Cas9-Target 1 (T1) or CRISPR/Cas9-Target 2 (T2) transfected Hep3B cells were cultured under normoxic (N) conditions or exposed to hypoxia (H) for 8 h. Protein masses are indicated in kDa. **b** Densitometry of ARNT protein level (corresponding to **a**) normalised to Lamin A/C. Values are presented as mean ± SEM of n = 4 independent experiments

To elucidate the role of the genomic DNA sequence designated as Region 3 for the cellular capability to elevate ARNT in hypoxia Hep3B cells were transiently transfected with CRISPR/Cas9-Target 1 and CRISPR/Cas9-Target 2 constructs respectively. Subsequently cells were exposed to hypoxia (3% O_2_) for 8 h or maintained in normoxia followed by Western blot analysis. As shown in Fig. 3a, HIF-1α was induced in hypoxic Hep3B cells compared to appropriate normoxic counterparts. However, the accumulation of HIF-1α was less pronounced in hypoxic CRISPR/Cas9-Target 1 transfected cells.

Unexpectedly, ARNT protein expression declined in normoxic CRISPR/Cas9-Target 1 and -Target 2 transfected cells (Fig. 3). Whereas, an elevated ARNT level was detected in untransfected Hep3B cells exposed to hypoxia as expected. Most important, ARNT expression was still inducible in hypoxic genome-edited cells but the level was clearly below the amount detected in untransfected counterparts. Due to the fact that HIF-1α was affected in CRISPR/Cas9-Target 1 transfected cells too, only CRISPR/Cas9-Target 2 was used for further experiments.

**Fig. 4:**
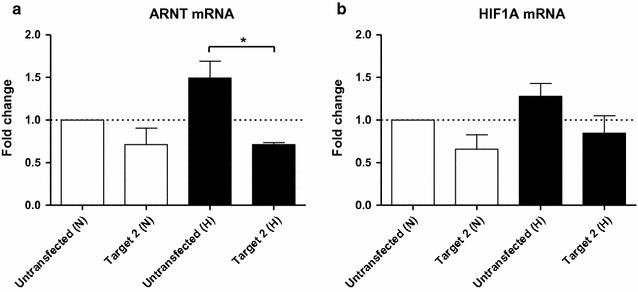
qRT-PCR analysis of CRISPR/Cas9 genome-edited Hep3B cells cultured in Lumox^®^ gas permeable petri-dishes. **a** ARNT mRNA- and **b** HIF1A mRNA expression were measured using TaqMan^®^ Gene Expression Assays under normoxic and hypoxic (3% O_2_, 5 h) conditions. Values are presented as mean ± SEM of n = 3 independent experiments. *N* normoxia, *H* hypoxia

The hypoxic elevation of ARNT in Hep3B cells was demonstrated on both mRNA and protein levels [6, 10, 12] and is driven by HIF-1α [12]. However, the effect on mRNA synthesis was less pronounced [12]. In order to test whether genome editing of the HIF-1α and ARNT enriched site might affect ARNT mRNA expression too, cells were transfected with the CRISPR/Cas9-Target 2 construct and analysed by qRT-PCR after hypoxic exposure (3% O_2_, 5 h). In addition, Lumox^®^ gas permeable petri-dishes were used as an attempt to strengthen the effect. As shown in Fig. 4a, ARNT mRNA was induced in untransfected hypoxic Hep3B cells as compared to normoxic control cells. Genome editing using CRISPR/Cas9-Target 2 decreased ARNT mRNA expression in normoxia and prevented hypoxic ARNT induction. However, no significant effects of genome editing on HIF-1α mRNA were observed (Fig. 4b).

In summary, these findings confirm the ChIP results and elucidate an important regulatory role of this genomic region regarding normoxic and hypoxic ARNT expression.

### Reduced ARNT expression in genome-edited cells inhibits HIF signalling

**Fig. 5:**
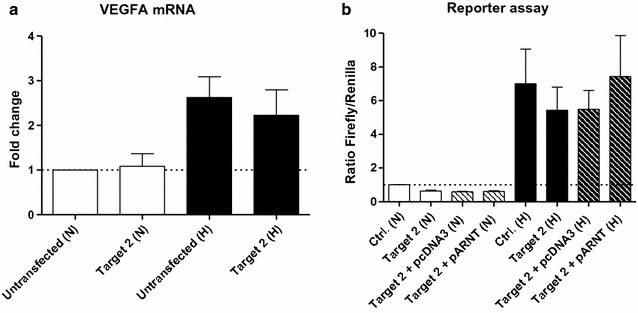
Effects of genome editing on HIF target gene expression. **a** VEGFA mRNA expression in CRISPR/Cas9 genome edited Hep3B cells cultured in Lumox^®^ gas permeable petri-dishes (corresponding to Fig. 4). Values are presented as mean ± SEM of n = 3 independent experiments. **b** Reporter gene assay. Ratios of *Firefly/Renilla* luciferase activity were measured in normoxic/hypoxic Hep3B cells after 8 h and normalised to normoxic control cells (Ctrl. (N)). Values are presented as mean ± SEM of n = 3 independent experiments. *N* normoxia, *H* hypoxia

Recently it was demonstrated by our group that ARNT acts as a limiting factor in hypoxic Hep3B cells [12]. Therefore, it was assumed that a decline in ARNT expression in genome-edited cells (Figs. 3, 4) might inhibit HIF signalling. In order to test this hypothesis VEGFA mRNA was measured in CRISPR/Cas9-Target 2 transfected cells and compared to appropriate controls under normoxic and hypoxic conditions. As expected, VEGF mRNA expression was upregulated in untransfected cells exposed to hypoxia for 5 h (Fig. 5a). On the other hand, induction of this HIF target gene seemed to be slightly affected in hypoxic genome-edited cells. To further confirm this finding reporter gene assays were performed. Therefore, cells were co-transfected with a hypoxia-inducible *Firefly* luciferase reporter construct and a constitutive *Renilla* luciferase expression vector for normalisation. Moreover, cells were transfected with the CRISPR/Cas9-Target 2 construct in conjunction with an ARNT expression vector or the appropriate empty plasmid. As shown in Fig. 5b, pronounced reporter activation was observed in control cells cultured under hypoxic conditions for 8 h as compared to normoxic counterparts. In agreement with the previous finding, the induction of *Firefly* luciferase expression was reduced in hypoxic genome edited cells. Co-transfection of cells with the CRISPR/Cas9-Target 2 construct and the pcDNA3 empty vector also impaired HIF signalling which indicates a lack of competition between both plasmids. Most important, overexpression of ARNT in genome-edited cells rescued the reporter activity in hypoxia.

Taken together, these results are in line with our previous report [12] and further confirm ARNT as being a limiting factor in this model.

### Recruitment of HIF-1α and ARNT to the *ARNT* promoter is not abolished by CRISPR/Cas9-Target 2 genome editing

**Fig. 6:**
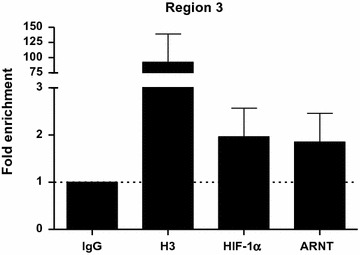
ChIP assays of genome-edited Hep3B cells exposed to hypoxia (3% O_2_, 5 h). Values are presented as mean ± SEM of n = 3 independent experiments. *IgG* normal rabbit IgG, *H3* histone H3

The results presented above demonstrate that genome editing using the CRISPR/Cas9-Target 2 construct impairs HIF signalling (Fig. 5). Moreover, this target sequence encompass two HAS elements within the *ARNT* promoter (Fig. 2a). Therefore, it was hypothesised that genome editing might prevent HIF-1α and ARNT recruitment under hypoxic conditions. To test this assumption genome-edited Hep3B cells were exposed to hypoxia for 5 h followed by ChIP analysis. As shown in Fig. 6, both transcription factors were still detected within the genomic DNA sequence designated as Region 3.

## Discussion

The induction of ARNT in hypoxia is a cell-specific attribute observed in tumour cells [2]. Until now two major advantages of an elevated ARNT expression level were revealed. Recently it was shown by our group that ARNT overexpression confers a radioresistant phenotype in tumour cells (including Hep3B) [11]. Moreover, it was demonstrated that ARNT upregulation under oxygen deprivation was mediated by its binding partner HIF-1α in two different cell lines (including Hep3B) [9, 12]. This non-canonical regulatory relationship constitutes a feed-forward loop leading to augmented HIF signalling in Hep3B cells [12]. By using this model, a transcriptional regulatory relationship between HIF-1α and ARNT was discovered [12]. However, whether this is the outcome of a direct or an indirect mechanism remained unclear. A direct regulation involves the binding of HIF-1α to the *ARNT* promoter whereas an indirect mechanism might be mediated by HIF-regulated factors (e.g., transcription factors, miRNAs, chromatin modifiers) [12]. Therefore, the aim of the present study was to test the hypothesis whether HIF-1α might be recruited to the *ARNT* promoter under oxygen deprivation.

Indeed, ChIP assays revealed the binding of HIF-1α at two distinct loci approximately 300–550 bp upstream of the *ARNT* transcription start site. Interestingly, ARNT was detected as well within the same regions. Noteworthy, the results show that ARNT is recruited to its own promoter even under normoxia which indicates a HIF-1α-independent event, at least partly. However, it is known that dimerisation of HIF proteins is strictly required for DNA binding [5]. This raises the question concerning the putative binding partner of ARNT under normoxic conditions. In general, all Per-ARNT-Sim proteins can bind to each other via PAS domain interactions [1, 15]. This includes the formation of ARNT homodimers which have been described in the literature [16]. Theoretically, an ARNT homodimer recruited to the *ARNT* gene promoter in normoxia might be replaced by the HIF-1 complex in hypoxia only by substitution of one ARNT subunit. Such a competition could explain the observation that ARNT mRNA and protein levels do not correlate in hypoxic Hep3B cells [12]. Indeed, a divergent ARNT mRNA and protein expression pattern was found in several other cell lines in hypoxia [10, 11]. Therefore, a reciprocal feedback regulation between ARNT protein level and de novo synthesis was already proposed by Wolff et al. [10].

The data presented in the current study implies that *ARNT* is a putative HIF-1 target gene in Hep3B cells. According to the definition, three criteria have to be fulfilled to designate a certain gene as a direct HIF target [17]: (1st) The gain or loss of HIF activity must correlate with target gene transcription under hypoxic conditions [17]. In our previous report we were able to demonstrate that hypoxia-dependent ARNT upregulation in Hep3B cells was mediated by HIF-1α. This regulatory relationship was shown on both mRNA and protein level [12]. (2nd) A *cis*-acting HRE must be identified in the gene which includes the 5′-RCGTG-3′ core sequence. Furthermore, the presence of this motif is required but not sufficient [17]. As shown in Fig. 1a, several HBS and HAS elements are located within 1200 bp upstream of the *ARNT* transcription start site. ChIP assays revealed the simultaneous binding of both HIF-1α and ARNT transcription factors at two distinct loci. This regions can be narrowed down to approximately 150–170 bp in length which is due to the selected PCR amplicon. Noteworthy, no canonical HRE (i.e., HBS and HAS in close proximity) was found in the sequence studied. In this regard, the spatial genome architecture has to be considered. An active chromatin configuration can be achieved when multiple regulatory elements are juxtaposed via looping [18]. In addition, there is evidence that most hypoxia-induced alterations in mRNA expression are cell-type specific [19]. The basal (i.e., normoxic) transcriptional activity of a certain locus is the major factor which governs the response to hypoxia. It was shown that HIF-1 binds preferentially to transcriptional active loci. Low affinity HIF binding sites might also be occupied by HIF-1 during prolonged hypoxia [19]. A recent study supports this concept [20]. It was revealed that preformed chromatin interactions between HIF-binding sites and distant gene promoters exist. The pre-existing chromatin architecture might define HIF target genes and contribute to cell-type specific hypoxic responses. In addition, these structures enable rapid gene activation in hypoxia [20]. However, chromosomal alterations are associated with the hallmarks of cancer [21]. For instance, structural abnormalities of chromosome 1, which harbours the *ARNT* gene [2], were frequently found in human HCC samples and human HCC cell lines such as Hep3B [22]. The 3rd requirement which has to be fulfilled to designate a certain gene as a direct HIF target assumes that disruption of HIF binding by mutagenesis causes a corresponding loss of oxygen regulated expression [17]. To confirm the results of the ChIP assays we deployed CRISPR/Cas9 genome editing in order to introduce specific DNA double strand breaks leading to insertions and deletions. This state-of-the-art technology enables new opportunities in biomedical research [23]. The CRISPR/Cas9 system is recommended to study the functional significance of genomic elements. In addition, this method can be used to perturb structural features which might provide a link between dysregulated chromatin architecture and cancer [18]. As shown in Figs. 3 and 4, genome editing resulted in a decreased normoxic ARNT expression level and inhibited hypoxia-dependent ARNT upregulation. Furthermore, by the use of CRISPR/Cas9-Target 2 a reduction of HIF signalling was observed (Fig. 5) which underscores the importance of this particular sequence. However, HIF-1α and ARNT were still recruited to the *ARNT* promoter in hypoxic genome-edited Hep3B cells (Fig. 6). Therefore, the 3rd requirement has not been proven.

The data presented in the current study suggests that ARNT controls its own expression. Such an autoregulation might explain conflicting evidence regarding the competition of HIF-α subunits and activated AhR for ARNT binding. Several studies support the concept of an antagonism between HIF and AhR signalling under oxygen deprivation and xenobiotic exposure [24–26]. In contrast, there is data which points to the opposite direction [27]. Therefore, it is reasonable to hypothesise whether hypoxic ARNT upregulation might overcome this competition and enables the full activation of both signalling pathways simultaneously under stressful conditions. In addition, this hypothesis can be considered to be the other way round. Theoretically, ARNT expression might be inducible by AhR signalling in certain cells. This assumption is supported by the presence of a 5′-TNGCGTG-3′ motif in Region 2 (not shown) which is known to be recognized by the AhR/ARNT heterodimer [28] and by the recruitment of ARNT to the same locus (Fig. 1).

However, the regulation of *ARNT* is poorly understood [29]. In addition to hypoxia-inducible ARNT expression found in different cell lines [9, 10], other conditions affecting the ARNT level are described in the literature. It was demonstrated that ARNT expression is inducible in various cell models by TNFα in a NF-κB dependent manner [29]. Moreover, a recent study revealed that ARNT is upregulated in vivo by dexamethasone via glucocorticoid receptor signalling [30]. These examples might suggest a more complex regulation of ARNT in response to a certain kind of stress or stimulus.

## Conclusions

**Fig. 7:**
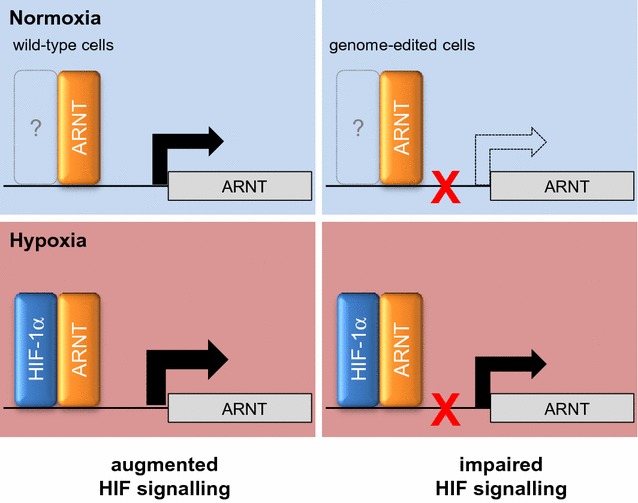
Proposed mechanism of hypoxia-inducible ARNT expression in Hep3B cells

Recently we discovered that HIF-1α elevates ARNT mRNA and protein expression in hypoxic Hep3B cells which constitutes a feed-forward loop leading to augmented HIF signalling [12]. The current study revealed a direct mechanism by which HIF-1α and ARNT are recruited to the *ARNT* gene promoter in hypoxia. The importance of this particular HIF-1α/ARNT enriched region for hypoxic inducibility of ARNT was confirmed by CRISPR/Cas9 genome editing. Genome-edited cells exhibited a reduced ARNT level and impaired HIF signalling (Fig. 7). Taken together, these findings indicate ARNT to be a putative HIF-1 target gene and a limiting factor in this model.

## Additional file

**Additional file 1: Figure S1.** Available CRISPR/Cas9 target sequences within Region 3. Selected sequences are highlighted.
