# Supplementary material for: ARNT is a potential direct HIF-1 target gene in human Hep3B hepatocellular carcinoma cells
Source: Cancer Cell Int. 2017 Aug 24;17:77. doi: 10.1186/s12935-017-0446-2 (PMC5571568; doi:10.1186/s12935-017-0446-2)
Supplement: Supplementary file 1 — Additional file 1: Figure S1. Available CRISPR/Cas9 target sequences within Region 3. Selected sequences are highlighted. [file 12935_2017_446_MOESM1_ESM.pdf]

region3

RGEN Target (5' to 3')[?]	Position[?]	Cleavage Position (%)[?]	Direction[?]	GC Contents (%, w/o PAM)[?]	Out-of-frame Score[?]	Mismatches[?]		
						0	1	2
GTAATCCCAGCTTCTTGGGAGGCTGAGG	3	4.7	-	56.0	N/A	331	39814	127274
CAGCCTCCCAAGAAGCTGGGATTACAGG	6	15.9	+	56.0	N/A	348	40249	129505
ATGCCTGTAATCCCAGCTTCTTGGGAGG	9	8.2	-	52.0	N/A	115	11512	48940
CGCATGCCTGTAATCCCAGCTTCTTGGG	12	10.0	-	52.0	N/A	23	2708	19362
GCGCATGCCTGTAATCCCAGCTTCTTGG	13	10.6	-	56.0	N/A	44	5832	26253
GATTACAGGCATGCGCCACCACGCCCGG	25	27.1	+	64.0	63.1	1749	14825	56983
AAAATACAAAATTAGCCGGGCGTGGTGG	40	26.5	-	44.0	64.3	2076	10914	20748
CTAAAAATACAAAATTAGCCGGGCGTGG	43	28.2	-	40.0	59.8	1930	10092	17239
CTCTACTAAAAATACAAAATTAGCCGGG	48	31.2	-	28.0	64.9	12706	18066	11880
TCTCTACTAAAAATACAAAATTAGCCGG	49	31.8	-	24.0	63.8	12454	11593	6090
GCTAATTTTGTATTTTTAGTAGAGACGG	52	42.9	+	24.0	69.3	14214	12978	6775
CTAATTTTGTATTTTTAGTAGAGACGGG	53	43.5	+	24.0	66.9	11636	18083	15116
TAATTTTGTATTTTTAGTAGAGACGGGG	54	44.1	+	24.0	62.9	6590	19641	70951
TAGTAGAGACGGGGTTTCGCCATGTTGG	68	52.4	+	52.0	61.0	2727	44379	131540
GAGACGGGGTTTCGCCATGTTGGCCAGG	73	55.3	+	64.0	62.6	2532	36509	130603
CGGGGTTTCGCCATGTTGGCCAGGCTGG	77	57.6	+	68.0	70.7	2287	29726	111525
GGGTTCGAAACCAGCCTGGCCAACATGG	87	54.1	-	60.0	59.8	39	2174	41581
GTTGGCCAGGCTGGTTTCGAACCCCTGG	91	65.9	+	64.0	64.8	8	206	4607
TGTGGCCAGGGGTTCGAAACCAGCCTGG	96	59.4	-	64.0	65.7	1	8	395
AGGCTGGTTTCGAACCCCTGGCCACAGG	98	70.0	+	64.0	60.0	1	4	84
TTCGAACCCCTGGCCACAGGTGATCCGG	106	74.7	+	60.0	56.9	1	0	0
AGGCGGCCGGATCACCTGTGGCCAGGGG	112	68.8	-	72.0	65.3	1	0	4
GAGGCGGCCGGATCACCTGTGGCCAGGG	113	69.4	-	72.0	64.2	1	0	5
CGAGGCGGCCGGATCACCTGTGGCCAGG	114	70.0	-	76.0	60.0	1	1	67
CTGGCCACAGGTGATCCGGCCGCCTCGG	115	80.0	+	72.0	57.9	1	2	111
GAGGCCGAGGCGGCCGGATCACCTGTGG	119	72.9	-	76.0	56.4	19	2330	17610
CAGCACTTTGGGAGGCCGAGGCGGCCGG	130	79.4	-	72.0	53.7	142	34180	101616
CGGCCGCCTCGGCCTCCCAAAGTGCTGG	131	89.4	+	76.0	N/A	55	16960	64484
